# Differential Activation of TRP Channels in the Adult Rat Spinal Substantia Gelatinosa by Stereoisomers of Plant-Derived Chemicals

**DOI:** 10.3390/ph9030046

**Published:** 2016-07-28

**Authors:** Eiichi Kumamoto, Tsugumi Fujita

**Affiliations:** Department of Physiology, Saga Medical School, 5-1-1 Nabeshima, Saga 849-8501, Japan; fujitat@cc.saga-u.ac.jp

**Keywords:** TRPV1, TRPA1, TRPM8, plant-derived chemical, l-glutamate release, spinal dorsal horn, patch-clamp, rat

## Abstract

Activation of TRPV1, TRPA1 or TRPM8 channel expressed in the central terminal of dorsal root ganglion (DRG) neuron increases the spontaneous release of l-glutamate onto spinal dorsal horn lamina II (substantia gelatinosa; SG) neurons which play a pivotal role in regulating nociceptive transmission. The TRP channels are activated by various plant-derived chemicals. Although stereoisomers activate or modulate ion channels in a distinct manner, this phenomenon is not fully addressed for TRP channels. By applying the whole-cell patch-clamp technique to SG neurons of adult rat spinal cord slices, we found out that all of plant-derived chemicals, carvacrol, thymol, carvone and cineole, increase the frequency of spontaneous excitatory postsynaptic current, a measure of the spontaneous release of l-glutamate from nerve terminals, by activating TRP channels. The presynaptic activities were different between stereoisomers (carvacrol and thymol; (−)-carvone and (+)-carvone; 1,8-cineole and 1,4-cineole) in the extent or the types of TRP channels activated, indicating that TRP channels in the SG are activated by stereoisomers in a distinct manner. This result could serve to know the properties of the central terminal TRP channels that are targets of drugs for alleviating pain.

## 1. TRP Channels Involved in Nociceptive Transmission through Dorsal Root Ganglion Neurons

Cation-permeable transient receptor potential (TRP) channels expressed in dorsal root ganglion (DRG) neurons are involved in nociceptive transmission from the periphery (for a review see [1]). TRP channels, which are synthesized in the cell body of the DRG neuron, are transported to the peripheral and central terminals of the neuron by axonal transport. Among TRP channels involved in the nociceptive transmission, there are TRP vanilloid-1 (TRPV1), TRP ankyrin-1 (TRPA1) and TRP melastatin-8 (TRPM8) channels. The TRPA1 channel is found in a subset of rat DRG neurons in which it is co-expressed with the TRPV1, but not the TRPM8 channel [2,3]. They are activated by chemical substances and temperature (for reviews see [4,5]). For instance, in the peripheral terminal of the DRG neuron, the TRPV1 channel is activated by capsaicin (a natural pungent ingredient contained in red peppers), protons and noxious heat (>43 °C; [6]; for review see [7]); the TRPA1 channel by pungent compounds in mustard, cinnamon and garlic (allyl isothiocyanate (AITC), cinnamaldehyde and allicin, respectively), and noxious cold temperature (<17 °C; [2,8,9,10]); and the TRPM8 channel by menthol (a secondary alcohol contained in peppermint or other mint) and mild temperature (<25 °C; [11,12]). Such an activation depolarizes the membrane of the peripheral terminal, resulting in the production of action potentials. As a result, the nociceptive or temperature information is transferred to the spinal dorsal horn.

On the other hand, TRP channels in the central terminal of the DRG neuron are expressed in the superficial laminae of the dorsal horn, especially the substantia gelatinosa (SG; lamina II of Rexed; [13,14]). In support of this idea about TRP activation in the SG, many of plant-derived chemicals increase in SG neurons the frequency of spontaneous excitatory postsynaptic current (sEPSC), a measure of the spontaneous release of l-glutamate from nerve terminals, by activating TRPV1 channel [15,16,17,18], TRPA1 channel [19,20] and TRPM8 channel [21,22] (for a review see [23]). The SG neurons play a pivotal role in modulating nociceptive transmission from the periphery [24,25,26] and thus TRP channels in the SG are involved in its modulation. Their expressions in the SG have been shown by immunohistochemistry [27].

## 2. Spinal Substantia Gelatinosa Involved in Regulating Nociceptive Transmission

Nociceptive transmission in the SG is in origin not only monosynaptic from glutamatergic DRG neurons but also polysynaptic from glutamate-, GABA- and/or glycine-containing interneurons [28]. In support of the involvement of the SG in nociceptive transmission, a plastic change in glutamatergic inputs to SG neurons through DRG neurons occurred in hyperalgesic rats that were subject to either an intraplantar injection of complete Freund’s adjuvant [29] or ovariectomy [30]. Endogenous and exogenous analgesics, which exhibit antinociception when administrated intrathecally, hyperpolarize membranes of SG neurons and reduce the release of l-glutamate onto SG neurons from nerve terminals, both of which actions reduce the membrane excitability of the SG neurons [31]. For example, opioids ([32]; for a review see [33]), nociceptin [34,35], a GABA_B_-receptor agonist baclofen [36,37], a μ-opioid receptor agonist tramadol [38], norepinephrine [39], serotonin [30,40], adenosine ([41,42]; for review see [43]), somatostatin [44,45], dopamine [46,47] and galanin [48] hyperpolarized membranes of rat SG neurons. Inhibition of the release of l-glutamate from nerve terminals onto rat SG neurons was produced by opioids ([49]; for review see [33]), nociceptin [50,51], baclofen [36,52,53], the endocannabinoid anandamide [16,54], norepinephrine [55], serotonin [30,40], adenosine ([41,56,57]; for a review see [43]) and galanin [48,58].

## 3. TRP Channels in Nociception

Much evidence demonstrates that TRP channels play a role in transferring nociceptive information. For example, neuropathic pain and hyperalgesia developed following excessive or ectopical expression of TRPV1 channel in the central nervous system (CNS) and peripheral nervous system, in both animals and humans [59,60,61,62]. Hyperalgesia in inflammatory pain models was reduced in TRPV1-knockout mice [63]. Nerve growth factor, which mediates inflammatory pain, upregulated the expression of TRPV1 channel in rat DRG neurons by an action of the small GTPase Ras [64]. Excessive expression of TRPV1 channel in primary-afferent fibers occurred in disease states including inflammatory disease and irritable bowel syndrome [65,66]. Peripheral inflammation upregulated TRPV1 channel involved in the enhancement of spontaneous excitatory transmission in rat SG neurons [67]. When intrathecally administrated, a powerful TRPV1 agonist resiniferatoxin (RTX, a component contained in the dried latex of the cactus-like plant; [68]) produced a prolonged antinociceptive response in dogs with bone cancer [69], possibly owing to a desensitization of TRPV1 channel. Selective inhibition of TRPV1 channel attenuated bone cancer pain in the mouse [70]. TRPV1 channel, which is a potential treatment target for cancer pain, was expressed in neurons that transfer information of this type of pain (for a review see [71]). Intrathecal application of a TRPV1 antagonist AS1928370 resulted in an inhibition of mechanical allodynia in a mouse model of neuropathic pain [72].

The TRPA1 agonist cinnamaldehyde evoked spontaneous pain, and induced mechanical hyperalgesia and cold hypoalgesia following its application to the forearm skin of human volunteers [73]. Moreover, TRPA1 channel was over-expressed in rat DRG neurons following peripheral inflammation and nerve injury, and cold hyperalgesia produced by inflammation and nerve injury was accompanied by the activation of the TRPA1 but not the TRPM8 channel [74]. Alternatively, TRPA1 channel was excessively expressed in the mouse spinal cord and DRG after peripheral inflammation occurred as a result of intraplantar injection of complete Freund’s adjuvant; intrathecally-applied TRPA1 antagonist reversed hyperalgesia that occurred in mouse models of neuropathic pain [75].

TRPM8 channel is also involved in nociceptive transmission. Proudfoot et al. [76] have proposed the idea that TRPM8 activation in both peripheral and central terminals of adult rat DRG neurons produces the release of l-glutamate from its central terminal onto dorsal horn neurons expressing group II/III metabotropic glutamate receptors, the activation of which results in antinociception. In chronic constrictive nerve injury rat models, which exhibited cold allodynia in hindlimbs, compared with the sham group, TRPM8-immunoreactive DRG neurons increased in number, menthol-sensitive DRG neurons increased in number in neurons that responded to capsaicin, and membrane currents produced by menthol in DRG neurons enhanced in amplitude [77]. Kono et al. [78] have suggested an involvement of TRPM8 channel in acute peripheral hypersensitivity to cold in oxaliplatin (a third-generation platinum analog)-treated cancer patients. In adult mice, (−)-menthol and its derivative WS-12 (which activated TRPM8 channel in DRG neurons) inhibited acute thermal, capsaicin- and acrolein-induced nociceptive behavior in a manner sensitive to a nonselective opioid-receptor antagonist naloxone, suggesting an involvement of endogenous opioids in antinociception produced by TRPM8 activation [79].

In conclusion, the effects of the activation of TRPV1, TRPA1 or TRPM8 channel on nociceptive transmission appear to depend on the location of the TRP channels activated, i.e., the peripheral or central terminal of DRG neuron, or both of them. When antinociceptive drugs are administrated intrathecally, it would be necessary to consider the activation of TRP channels located in not only primary-afferent central terminal but also neuronal and glial cells in the CNS, because their expressions in the CNS have been reported [80,81].

## 4. Actions of Plant-Derived Stereoisomers on Spontaneous Excitatory Transmission in Substantia Gelatinosa Neurons

It is well-known that there is a difference among stereoisomers in their actions on voltage-gated ion channels and neurotransmitter receptors (see [82,83] for reviews). Activation of TRPA1 channel expressed in Chinese hamster ovary cells differs in efficacy between stereoisomers such as (+)-menthol and (−)-menthol [84]. In order to know whether such a difference between stereoisomers is seen in the SG, we examined the actions of plant-derived stereoisomers on spontaneous excitatory transmission in SG neurons with a focus on TRP activation by using transverse slice preparations dissected from the adult rat spinal cord [85].

### 4.1. Actions of Thymol and Carvacrol

**Figure 1:**
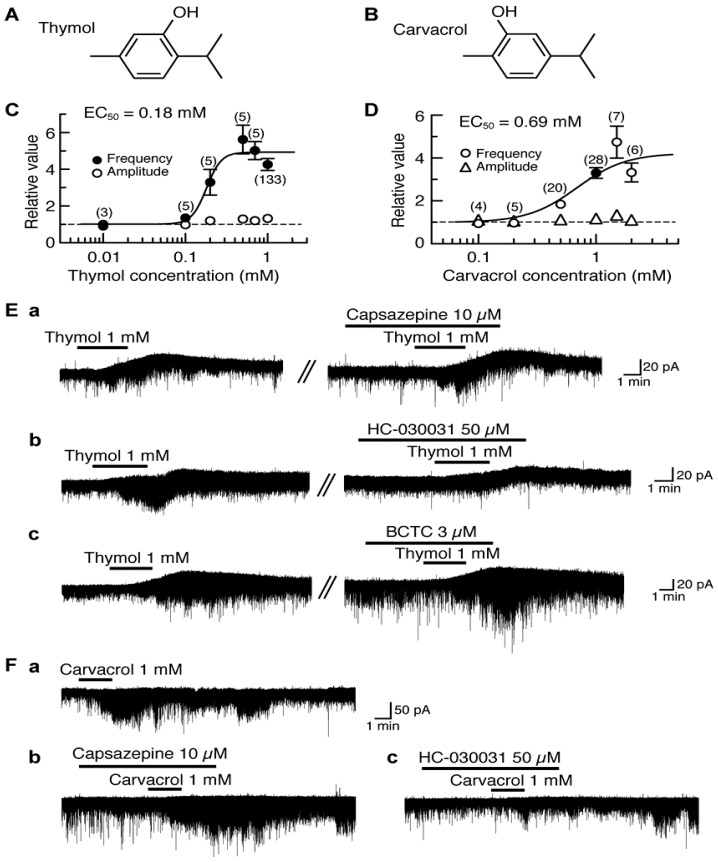
Effects of thymol and carvacrol on glutamatergic spontaneous excitatory transmission in rat substantia gelatinosa (SG) neurons. (**A**,**B**) The chemical structures of thymol (**A**) and carvacrol (**B**). (**C**,**D**) The frequency and amplitude of sEPSC under the action of thymol (**C**) or carvacrol (**D**), relative to those before drug superfusion, which were plotted against the logarithm of drug concentration. This thymol (carvacrol) effect was measured for 0.5 min around 5 min (3.5 min) after the beginning of its superfusion. The results in (**C**) were obtained from all neurons tested, while those in (**D**) were obtained from neurons where carvacrol (1 mM) increased sEPSC frequency > 5%. The continuous curves in (**C**) and (**D**) were drawn according to the Hill equation [half-maximal effective concentration (EC_50_) and Hill coefficient (n_H_) in (**C**) and (**D**): 0.18 mM, 4.9 and 0.69 mM, 2.1, respectively]. (**Ea**–**c**) Chart recordings showing sEPSCs and holding currents in the absence and presence of thymol in Krebs solution without (left) or with a TRPV1 antagonist capsazepine (**Ea**), a TRPA1 antagonist HC-030031 (**Eb**) or a TRPM8 antagonist BCTC (**Ec**; right). In each of (**Ea**)–(**Ec**), the right recording was obtained about 30 min after the left one from the same neuron. (**Ea**–**c**) Chart recordings showing sEPSCs in the absence and presence of carvacrol in Krebs solution without (**Fa**) and with capsazepine (**Fb**) or HC-030031 (**Fc**); these recordings were obtained from the same neuron at an interval of 30 min. In this and subsequent figures, value in parentheses indicates the number of neurons tested; each point with vertical bars represents the mean values and standard error of the mean (SEM); if the SEM of the values is less than the size of symbol, the vertical bar is not shown; control level (1) is indicated by horizontal dotted line; the duration of drug superfusion is shown by a horizontal bar above the chart recording. Holding potential (V_H_) = −70 mV. This research was originally published in [88,89].

Thymol (5-methyl-2-isopropylphenol, a compound where the cyclohexane ring of menthol is replaced by a benzene ring) differs only in the position of the -OH in the benzene ring from carvacrol (5-isopropyl-2-methylphenol; Figure 1A,B). Thymol and carvacrol are contained in thyme and oregano, respectively, and exhibit antinociception [86,87]. In all SG neurons tested, bath-applied thymol (1 mM) for 3 min increased the frequency of sEPSC with a small increase in its amplitude. The sEPSC frequency increase averaged to be 326% around 5 min (when a maximal effect was obtained) after the onset of thymol superfusion. Such a sEPSC frequency increase was concentration-dependent with the half-maximal effective concentration (EC_50_) value of 0.18 mM (Figure 1C). In 77% of the neurons tested, thymol (1 mM) produced an outward current having the averaged peak amplitude of 16 pA at the V_H_ of −70 mV (see Figure 1E); remaining neurons had no outward currents [88].

Carvacrol exhibited similar actions to those of thymol. In 22% of the SG neurons tested, carvacrol (1 mM) superfused for 2 min produced an outward current, which was not accompanied by a change in sEPSC frequency, at −70 mV. On the other hand, 11% of the SG neurons produced no change in holding currents while exhibiting sEPSC frequency increase (see Figure 1Fa). In 63% of the neurons, both of the outward current and sEPSC frequency increase were produced [89].

sEPSC frequency increase around 3.5 min (when a maximal effect was obtained) after the beginning of carvacrol superfusion averaged to be 262% with a small increase in its amplitude and the outward current had the averaged peak amplitude of 26 pA. Such a sEPSC frequency increase was concentration-dependent with the EC_50_ value of 0.69 mM (Figure 1D; [89]), a value larger than that of thymol. Thymol has an ability to activate TRP vanilloid-3 (TRPV3; [90]), TRPA1 [91] and TRPM8 channels [92] expressed in heterologous cells. On the other hand, carvacrol has been reported to activate TRPV3 and TRPA1 channels but not TRPV1 channel expressed in human embryonic kidney (HEK) or *Xenopus laevis* oocyte cells [90,91,93,94]. We next examined what types of TRP channel mediate the sEPSC frequency increases produced by thymol and carvacrol. The thymol activity was inhibited by a TRPA1 antagonist HC-030031 (50 μM; [95]) but not a TRPV1 antagonist capsazepine (10 μM; [96]) and a TRPM8 antagonist (4-(3-chloro-2-pyridinyl)-*N*-[4-(1,1-dimethyl-ethyl)phenyl]-1-piperazinecarboxamide, BCTC, 3 μM; [97]; Figure 1E). BCTC at this concentration was effective in inhibiting sEPSC frequency increase produced by menthol in adult rat SG neurons [27]. As with thymol, the carvacrol activity was resistant to capsazepine (10 μM) while being depressed by HC-030031 (50 μM; Figure 1F). These results indicate an involvement of TRPA1 channel in the presynaptic activities of thymol and carvacrol. As distinct from these presynaptic actions, the outward currents produced by thymol and carvacrol were insensitive to capsazepine (10 μM), BCTC (3 μM) and HC-030031 (50 μM), indicating no involvement of TRP channels ([88,89]; for example see Figure 1E). The carvacrol current was inhibited in 10 mM-K^+^ but not K^+^-channel blockers (5 mM tetraethylammonium and 0.1 mM Ba^2+^)-containing and 11.0 mM-Cl^−^ Krebs solution, indicating an involvement of tetraethylammonium- and Ba^2+^-insensitive K^+^ channels [89]. It remains to be examined what types of ion channel are involved in the thymol current.

### 4.2. Actions of (−)-Carvone and (+)-Carvone

**Figure 2:**
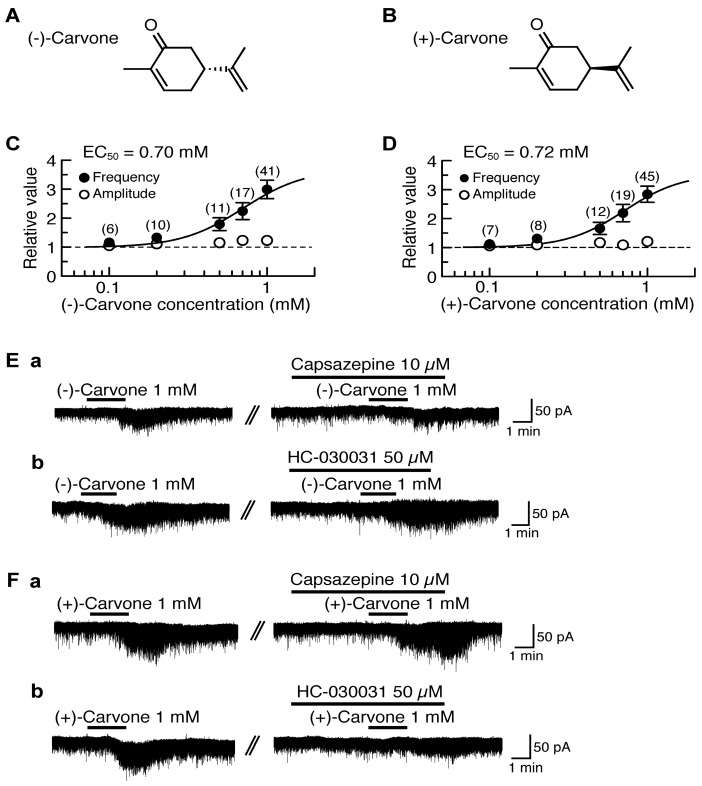
Effects of (−)-carvone and (+)-carvone on glutamatergic spontaneous excitatory transmission in rat SG neurons. (**A**,**B**) The chemical structures of (−)-carvone (**A**) and (+)-carvone (**B**). (**C**,**D**) The frequency and amplitude of sEPSC under the action of (−)-carvone (**C**) or (+)-carvone (**D**), relative to those before drug superfusion, which were plotted against the logarithm of drug concentration. This carvone effect was measured for 0.5 min around 3 min after the beginning of its superfusion. The continuous curves in (**C**) and (**D**) were drawn according to the Hill equation (EC_50_ and n_H_ in (**C**) and (**D**): 0.70 mM, 2.2 and 0.72 mM, 2.4, respectively). (**Ea,b,Fa,b**) Chart recordings showing sEPSCs in the absence and presence of (−)-carvone (**E**) or (+)-carvone (**F**) in Krebs solution without (left) and with capsazepine (**Ea,Fa**) or HC-030031 (**Eb,Fb**; right). In each of (**Ea,b,Fa,b**), the right recording was obtained about 20 min after the left one from the same neuron. V_H_ = −70 mV. This research was originally published in [101].

(−)-Carvone [(−)-2-methyl-5-(1-methylethenyl)-2-cyclohexenone; Figure 2A] contained in spearmint increased intracellular Ca^2+^ concentration in rat DRG neurons in a manner sensitive to capsazepine, indicating an involvement of TRPV1 channel [98]. A similar action of (−)-carvone was observed in HEK293 cells expressing human TRPV1 channel [98]. (+)-Carvone (a stereoisomer of (−)-carvone; Figure 2B) contained in caraway has been shown to have actions different from those of (−)-carvone in mouse locomotive [99] and anticonvulsive activities [100]. We examined the effects of (−)-carvone and (+)-carvone on glutamatergic spontaneous excitatory transmission with a focus on TRP activation. (−)-Carvone and (+)-carvone (each 1 mM) superfused for 2 min increased the frequency of sEPSC with a slight increase in its amplitude. Their sEPSC frequency increases averaged to be 299% and 284%, respectively, around 3 min (when a maximal effect was obtained) after the beginning of its superfusion. Such presynaptic activities of (−)-carvone and (+)-carvone were concentration-dependent with the EC_50_ values of 0.70 mM and 0.72 mM, respectively (Figure 2C,D).

(−)-Carvone and (+)-carvone (each 1 mM) did not produce any outward currents, as different from thymol and carvacrol. About 40% of the SG neurons tested produced a small inward current following the application of (−)-carvone or (+)-carvone [101], as seen by many kinds of TRPV1 agonists (capsaicin and RTX; [15,18]) and TRPA1 agonists (AITC, cinnamaldehyde and allicin; [19]).

The sEPSC frequency increase produced by (−)-carvone was resistant to HC-030031 (50 μM) while being inhibited by capsazepine (10 μM; Figure 2Ea,b). On the other hand, the sEPSC frequency increase produced by (+)-carvone was inhibited by HC-030031 (50 μM) while being resistant to capsazepine (10 μM; Figure 2Fa,b). These results indicate that (−)-carvone and (+)-carvone activate TRPV1 and TRPA1 channels, respectively, in the SG [101].

### 4.3. Actions of 1,8-Cineole and 1,4-Cineole

**Figure 3:**
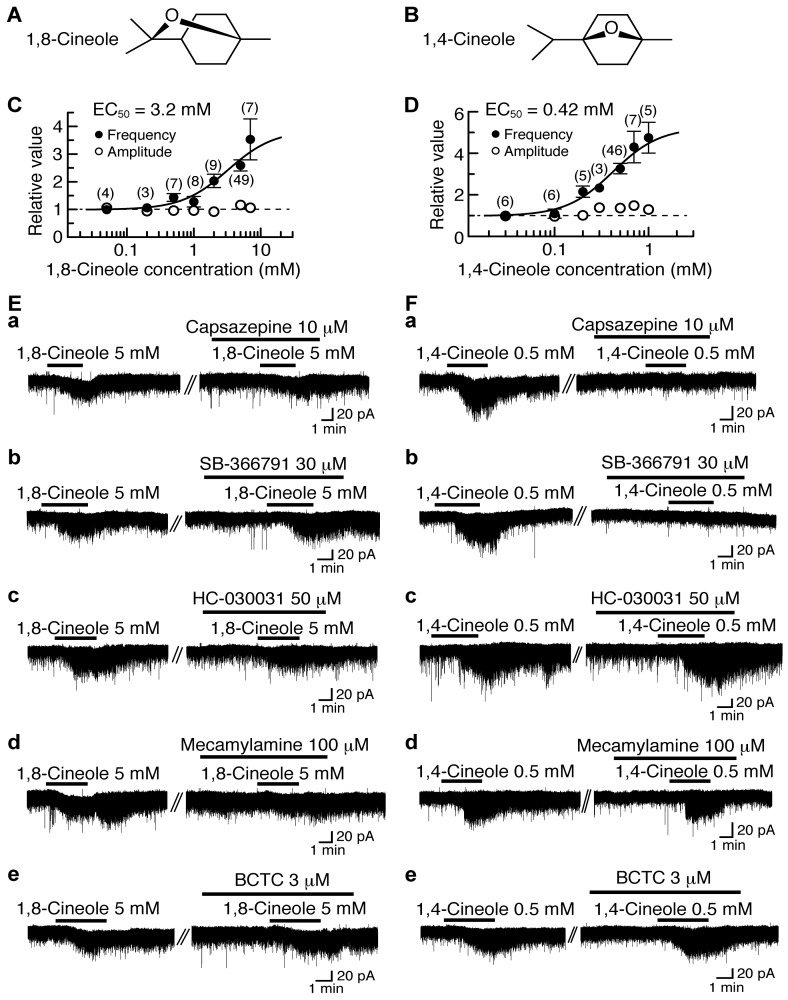
Effects of 1,8-cineole and 1,4-cineole on glutamatergic spontaneous excitatory transmission in rat SG neurons. (**A**,**B**) The chemical structures of 1,8-cineole (**A**) and 1,4-cineole (**B**). (**C**,**D**) The frequency and amplitude of sEPSC under the action of 1,8-cineole (**C**) or 1,4-cineole (**D**), relative to those before drug superfusion, which were plotted against the logarithm of drug concentration. This cineole effect was measured for 0.5 min around 3.5 min after the addition of its drug. The continuous curves in (**C**) and (**D**) were drawn according to the Hill equation (EC_50_ and n_H_ in (**C**) and (**D**): 3.2 mM, 1.3 and 0.42 mM, 1.7, respectively). (**Ea**–**e,Fa**–**e**) Chart recordings showing sEPSCs in the absence and presence of 1,8-cineole (**E**) or 1,4-cineole (**F**) in Krebs solution without (left) and with capsazepine (**a**), SB-366791 (**b**), HC-030031 (**c**), mecamylamine (**d**) or BCTC (**e**; right). In each of (**Ea**–**e,Fa**–**e**), the right recording was obtained about 20 min after the left one from the same neuron. V_H_ = −70 mV. This research was originally published in [27].

1,8-Cineole (1,3,3-trimethyl-2-oxabicylo[2.2.2]octane; Figure 3A), which is present in eucalyptus and rosemary, has various actions including antinociception [102]. As a minor component of plant extracts containing 1,8-cineole, there is its stereoisomer 1,4-cineole (1-methyl-4-(1-methylethyl)-7-oxabicyclo[2.2.1]heptane; Figure 3B), which has on plant species an action which is different from that of 1,8-cineole [103].

1,8-Cineole and 1,4-cineole have an ability to activate TRPM8 channel expressed heterologously in *Xenopus* oocytes [11] or HEK293 cells [104,105], although their efficacies are much less than that of the TRPM8 agonist menthol. We examined the effects of 1,8-cineole and 1,4-cineole on glutamatergic spontaneous excitatory transmission with a focus on TRP activation. As with (−)-carvone and (+)-carvone, bath-applied 1,8-cineole and 1,4-cineole for 3 min increased the frequency of sEPSC with a small increase in its amplitude. The sEPSC frequency increases produced by 1,8-cineole and 1,4-cineole (5 mM and 0.5 mM, respectively) averaged to be 159% and 226%, respectively, around 3.5 min (when a maximal effect was obtained) after the beginning of its superfusion. Such presynaptic actions of 1,8-cineole and 1,4-cineole were concentration-dependent with the EC_50_ values of 3.2 mM and 0.42 mM, respectively (Figure 3C,D).

The presynaptic activities of 1,8-cineole and 1,4-cineole were not accompanied by the production of outward current, as different from those of thymol and carvacrol. As with many kinds of TRPV1 and TRPA1 agonists [15,18,19] including (−)-carvone and (+)-carvone, 1,8-cineole and 1,4-cineole produced a small inward current (Figure 3E,F; [27]).

The presynaptic action of 1,8-cineole was inhibited by HC-030031 (50 μM) and another TRPA1 antagonist mecamylamine (100 μM; which is known to be also a nicotinic acetylcholine-receptor antagonist [106]) while being resistant to capsazepine (10 μM) and another TRPV1 antagonist SB-366791 (30 μM; [67]; Figure 3Ea–d). On the contrary, 1,4-cineole’s one was depressed by capsazepine (10 μM) and SB-366791 (30 μM) while being insensitive to HC-030031 (50 μM) and mecamylamine (100 μM; Figure 3Fa–d). BCTC (3 μM) did not affect the activities of 1,8-cineole and 1,4-cineole (Figure 3Ee,Fe). These results indicate that 1,8-cineole and 1,4-cineole activate TRPA1 and TRPV1 channels, respectively, in the SG.

## 5. Activation by Plant-Derived Stereoisomers of TRP Channels in the Substantia Gelatinosa in a Different Manner

Thymol and carvacrol, which are distinct only in the position of the -OH in the benzene ring (Figure 1A,B), activated the TRPA1 channel with EC_50_ values which differ four-fold from each other. Optic isomers, (–)-carvone and (+)-carvone (Figure 2A,B), activated TRPV1 and TRPA1 channels, respectively, with almost the same EC_50_ value. 1,8-Cineole and 1,4-cineole, which are different in the placement of the oxygen bridge (Figure 3A,B; where there is a free dimethyl side chain in 1,4-cineole but not 1,8-cineole [103]), activated TRPA1 and TRPV1 channels, respectively, with EC_50_ values eight-fold different from each other. The TRPV1 and TRPA1 activations resulted in an increase in spontaneous l-glutamate release from nerve terminals onto SG neurons. These results indicate that TRP channels in the SG have an ability to discriminate plant-derived stereoisomers from each other.

The stereoisomers mentioned in this review article are not endogenous ones that act on TRP channels located in the central terminals of DRG neurons under physiological conditions. There are several candidates for endogenous substances that activate TRP channels. For example, endogenous agonists for TRPV1 channel include endocannabinoids such as anandamide and lipoxygenase metabolites, which have structures similar to that of capsaicin which is not produced endogenously ([107,108]; for reviews see [7,109]). TRPV1 channel in the SG did not appear to be activated by anandamide [54] while anandamide-transport inhibitor AM404 activated the SG TRPV1 channel [110]. As candidates of endogenous TRPA1 activators, there is a potent and systemically active inhibitor of fatty acid amide hydrolase, 3′-carbamoylbiphenyl-3-yl cyclohexylcarbamate (URB597; [111]), a cyclopentane prostaglandin D_2_ metabolite (15-deoxy-Δ^12,14^-prostaglandin J_2_; [112]) or bradykinin [113]. To our knowledge, endogenous agonists for TRPM8 channel do not appear to be surely identified. Testosterone (a steroid hormone from the androgen group) has been recently reported to activate TRPM8 channel [114,115]. Although endogenous stereoisomers for TRP activation do not appear to be available, our findings about stereoisomers could serve to know the properties of the central terminal TRP channels.

Many of the properties of the TRP channels have been examined in the cell body of the primary-afferent neuron and in heterologous cell expressing the TRP channels. We have found out that a local anesthetic lidocaine, which acts on TRPV1 channel [116] and by a less extent on TRPA1 channel [117] in the cell body of primary-afferent neuron, activates TRPA1 but not TRPV1 channel in its central terminal [118]. The central terminal TRPV1 channel was activated by piperine (a pungent component of black pepper; [119]) but not olvanil (the synthetic oleic acid homologue of capsaicin; [120]), both of which compounds activated TRPV1 channel in the cell body of primary-afferent neuron [121,122]. Vanilloid compounds, eugenol (contained in clove) and zingerone (a pungent component of ginger), that reportedly activated TRPV1 channel in the cell body of primary-afferent neuron [121,123] were shown to activate the central terminal TRPA1 but not TRPV1 channel [124,125]. Based on their findings, we have proposed the idea that TRP channels located in the cell body and central terminal of the primary-afferent neuron have properties different from each other [23]. Such a difference may be due to a distinction between cell body and central terminal TRPA1 channels in terms of a functional interaction of TRPA1 channel and toll-like receptor-7 ([126]; see [125] for the other possibilities). It remains to be examined whether TRP channels in the cell body of DRG neuron are activated by plant-derived stereoisomers in a distinct manner.

TRPA1 channel in the central terminal of DRG neuron in the SG is activated by many plant-derived chemicals. Very recently, we have reported that citral, a mixture of geranial and neral, which is contained in lemongrass, activates TRPA1 channel in the SG [127]. Table 1 summarizes available values of EC_50_ for plant-derived chemicals in activating TRPV1 and TRPA1 channels in the adult rat SG. Their efficacy sequence for the TRPA1 activation was thymol (EC_50_ = 0.18 mM) > citral (0.58 mM) ≥ carvacrol (0.69 mM) ≥ (+)-carvone (0.72 mM) > zingerone (1.3 mM) > 1,8-cineole (3.2 mM) ≥ eugenol (3.8 mM). This result could serve to know the property of central terminal TRPA1 channel that is a target of drugs for alleviating pain together with the above-mentioned results of stereoisomers.

TRP activation in the SG is generally thought to be involved in nociception [1], because the enhancement of the spontaneous release of l-glutamate from nerve terminals onto SG neurons as a result of the TRP activation increases an excitability of the SG neurons, an action different from those of analgesic substances (see Section 2). However, antinociception produced by the intrathecal administration of acetaminophen has been attributed to TRPA1 activation in the superficial spinal dorsal horn [128]. AITC inhibited current responses recorded from SG neurons by using the in vivo patch-clamp technique [129] in response to pinch stimuli given to the skin [130]. It remains to be addressed whether TRPA1 activation in the SG results in nociception or antinociception.

Although the present review article mentions the actions of plant-derived stereoisomers on excitatory transmission, the regulation of nociceptive transmission in the SG is due to a modulation of not only excitatory but also GABAergic and/or glycinergic inhibitory transmissions [25,131,132]. It is possible that a modulation of inhibitory transmission by TRP activation is involved in nociceptive transmission. There is much evidence supporting the idea that inhibitory transmission enhancement in the spinal dorsal horn results in antinociception. First, the lack of GABA-synthesizing enzyme [133,134] and also reduction in the expression of K^+^-Cl^−^ exporter KCC2, which causes inhibitory synaptic response to be excitatory [135], in the rat spinal dorsal horn leaded to nociception. Second, peripheral inflammation resulted in a reduced glycinergic transmission in rat spinal lamina I neurons [136]. Third, an increase in endogenous glycine due to glycine transporter-1 blockade produced an inhibitory effect on spinal nociceptive transmission ([137]; for reviews see [138,139]). Fourth, endogenous analgesics, acetylcholine, norepinephrine and serotonin, enhanced GABAergic and glycinergic inhibitory transmissions [140,141,142,143,144,145]. It appears to depend on the agonists used to activate TRP channels how TRP activation affects spontaneous inhibitory transmission in SG neurons. AITC and zingerone enhanced inhibitory transmission [19,125] while capsaicin and cinnamaldehyde did not [15,20]. It remains to be examined how plant-derived stereoisomers affect spontaneous inhibitory transmission.

Itch is partly similar to pain in the neuronal pathway and receptors involved, although they are distinct from each other in many points of neuronal transmissions. There is much evidence supporting the idea that TRPV1 and TRPA1 channels are involved in itch. For instance, a topical application of capsaicin to the skin produced itch [146,147]. It is thus likely that TRP channels located in the central terminal of a pruriceptive primary-afferent neuron are involved in the modulation of itch sensation. Although proteinase-activated receptor (PAR)-2 in the peripheral terminal of a primary-afferent neuron is involved in the production of itch [148,149], other types of PAR may play a role in modulating pruriceptive transmission in the spinal dorsal horn. Fujita et al. [150] have reported that PAR-1 activation presynaptically increases spontaneous excitatory transmission in adult rat SG neurons. The findings about plant-derived stereoisomers, mentioned in this review article, may serve to know how to modulate itch transmission in the spinal dorsal horn.

There are known to be species differences on the pharmacology of TRP channels. For example, Jordt and Julius [151] have found out that chicken TRPV1 channel has a much less sensitivity to capsaicin than rat one. Rat and human TRPV1 channels were about 100-fold more sensitive to capsaicin than rabbit one [152]. 4-Methyl-*N*-[2,2,2-trichloro-1-(4-nitro-phenylsulfanyl)-ethyl]-benzamide activated rat TRPA1 channel while blocking human TRPA1 activation by reactive and nonreactive agonists [153]. Caffeine inhibited human TRPA1 channel but activated mouse TRPA1 channel [154]. A967079 blocked mammalian TRPA1 channel [155], but failed to inhibit chicken TRPA1 channel [156]. It remains to be investigated whether a difference in TRP activation among plant-derived stereoisomers is seen in animal species other than rats.

## 6. Conclusions

Plant-derived chemical stereoisomers, i.e., thymol and carvacrol, (−)-carvone and (+)-carvone, or 1,8-cineole and 1,4-cineole, activated TRP channels in the SG in a manner different from each other in the extent and the types of TRP channels activated. TRP channels expressed in the central terminals of DRG neurons are thus suggested to have an ability to discriminate stereoisomers.

## Figures and Tables

**Table 1:** EC_50_ values for plant-derived chemicals in activating TRPV1 and TRPA1 channels in the adult rat SG.

Plant-Derived Chemicals	TRPV1 (mM)	TRPA1 (mM)	References
Resiniferatoxin	2.1 × 10^−4^	−	[18]
Piperine	0.052	−	[120]
Eugenol	−	3.8	[124]
Zingerone	−	1.3	[125]
(−)-Carvone	0.70	−	[101]
(+)-Carvone	−	0.72	[101]
Carvacrol	−	0.69	[89]
Thymol	−	0.18	[88]
1,8-Cineole	−	3.2	[27]
1,4-Cineole	0.42	−	[27]
Cital	−	0.58	[127]
